# House dust mite-specific immunotherapy and tropomyosin sensitizations: harm or benefit for patients?

**DOI:** 10.1186/2045-7022-3-S3-P44

**Published:** 2013-07-25

**Authors:** B Pevec, M Radulovic Pevec, A Stipic Markovic, I Batista

**Affiliations:** 1 Department of Clinical Immunology, Pulmology, and Rheumatology, Clinical Hospital Sveti Duh, Zagreb, Croatia; 2 Immunological Laboratory, Clinical Hospital Sveti Duh, Zagreb, Croatia

## Background

Allergenic tropomyosins are highly cross-reactive panallergens found in various invertebrates. Sensitized patients can develop allergic responses ranging from oral allergy syndrome to severe anaphylactic reactions after the ingestion of tropomyosin-containing food. The usual routs of sensitization to tropomyosin include the airborne exposure to various mites and cockroaches and the oral intake of crustaceans and mollusks. There were also reports of patients with combined mite and seafood allergy who had experienced a worsening of food allergy symptoms during house dust mite-specific immunotherapy (HDM-SCIT). Therefore, a possibility that mite tropomyosin present in allergen extracts can cross-sensitize patients receiving HDM-SCIT and thus induce food allergy is still investigated.Our aim was to assess the occurrence and the clinical relevance of tropomyosin sensitizations in HDM-SCIT-treated patients.

## Methods

We studied 56 HDM-allergic patients treated with mite extract-SCIT. All of them were initially screened for sIgE to mite tropomyosin (rDer p 10) before and after SCIT. In those with a positive result we also monitored the dynamics of sIgE to both mite and shrimp (rPen a 1) tropomyosins at five time points: prior to treatment, after initial therapy (~4 months), 1 and 2 years after starting therapy, and after its completion. The levels of sIgE were measured by the CAP-System FEIA.

## Results

sIgE to tropomyosin was found in only 5 patients. Of those, 3 expressed low and clinically irrelevant levels of sIgE to Der p 10 during SCIT, while sIgE to Pen a 1 was not found at all. The remaining 2 patients expressed sIgE to both tropomyosins. In the first, initial increase and subsequent decrease resembled the dynamics of IgE-antibodies usually seen in SCIT patients, while clinically he had never expressed an allergic reaction to tropomyosin-containing foods. In the other, who used to develop oral allergy syndrome after consuming squids or shrimps before SCIT, a decrease of sIgE to both tropomyosins resulted in the complete loss of his reactivity toward seafood.

## Conclusion

Induction of clinically relevant tropomyosin sensitizations is an unlikely consequence of HDM-SCIT.In some cases of combined mite and seafood allergy the treatment may even lead to the improvement of food allergy symptoms.The levels of sIgE to tropomyosins might be useful in monitoring, especially if their cutoff values predictive of the reaction severity could be determined.

## Disclosure of interest

None declared.

